# Delayed sampling of intraoperative parathormone may be unnecessary during parathyroidectomy in kidney-transplanted and dialysis patients

**DOI:** 10.1590/2175-8239-JBN-2020-0108

**Published:** 2021-01-15

**Authors:** Andre Albuquerque Silveira, Marilia D'Elboux Guimarães Brescia, Climério Pereira do Nascimento, Sergio Samir Arap, Fabio Luiz de Menezes Montenegro

**Affiliations:** 1 Universidade de São Paulo, Faculdade de Medicina, Hospital das Clínicas HCFMUS, Departamento de Cirurgia, Cirurgia de Cabeça e Pescoço, São Paulo, SP, Brasil.

**Keywords:** Parathyroidectomy, Parathyroid Hormone, Intraoperative Monitoring, Paratireoidectomia, Hormônio Paratireóideo, Monitorização Intraoperatória

## Abstract

**Introduction:**

Some authors advise in favor of delayed sampling of intraoperative parathormone testing (ioPTH) during parathyroidectomy in dialysis and kidney-transplanted patients. The aim of the present study was to evaluate the intensity and the role of delayed sampling in the interpretation of ioPTH during parathyroidectomy in dialysis patients (2HPT) and successful kidney-transplanted patients (3HPT) compared to those in single parathyroid adenoma patients (1HPT).

**Methods:**

This was a retrospective study of ioPTH profiles in patients with 1HPT, 2HPT, and 3HPT operated on in a single institution. Samples were taken at baseline ioPTH (sampling at the beginning of the operation), ioPTH-10 min (10 minutes after excision of the parathyroid glands), and ioPTH-15 min (15 minutes after excision of the parathyroid glands). The values were compared to baseline.

**Results:**

Median percentage values of ioPTH compared to baseline (100%) were as follows: 1HPT, ioPTH-10 min = 20% and ioPTH-15 min = 16%; 2HPT, ioPTH-10 min = 14% and ioPTH-15 min = 12%; 3HPT, ioPTH-10 min = 18% and ioPTH-15 min = 15%.

**Discussion:**

The reduction was equally effective at 10 minutes in all groups. In successful cases, ioPTH decreases satisfactorily 10 minutes after parathyroid glands excision in dialysis and transplanted patients, despite significant differences in kidney function. The postponed sampling of ioPTH appears to be unnecessary.

## Resumo

**Introdução:**

Alguns autores aconselham a favor de se fazer uma amostragem tardia de teste de paratormônio intraoperatório (PTHIO) durante paratireoidectomia em pacientes transplantados renais e em diálise. O objetivo do presente estudo foi avaliar a intensidade e o papel da amostragem tardia na interpretação do PTHIO durante paratireoidectomia em pacientes em diálise (2HPT) e pacientes com transplante renal bem sucedido (3HPT) em comparação com aqueles em pacientes com adenoma único de paratireoide (1HPT).

**Métodos:**

Este foi um estudo retrospectivo dos perfis de PTHIO em pacientes com 1HPT, 2HPT, e 3HPT operados em uma única instituição. Foram coletadas amostras de PTHIO basal (amostragem no início da operação), PTHIO-10 min (10 minutos após a excisão das glândulas paratireoides), e PTHIO-15 min (15 minutos após a excisão das glândulas paratireoides). Os valores foram comparados aos resultados basais.

**Resultados:**

Os valores percentuais medianos do PTHIO em comparação aos basais (100%) foram os seguintes: 1HPT, PTHIO-10 min = 20% e PTHIO-15 min = 16%; 2HPT, PTHIO-10 min = 14% e PTHIO-15 min = 12%; 3HPT, PTHIO-10 min = 18% e PTHIO-15 min = 15%.

**Discussão:**

A redução foi igualmente eficaz aos 10 minutos em todos os grupos. Em casos de sucesso, o PTHIO diminui satisfatoriamente 10 minutos após a excisão das glândulas paratireoides em pacientes em diálise e transplantados, apesar das diferenças significativas na função renal. A amostragem tardia de PTHIO parece ser desnecessária.

## Introduction

There is an increased risk of death in dialysis patients with secondary hyperparathyroidism (2HPT)^1^. Successful parathyroidectomy (PTx) improves survival^2^^-^ 4 and quality of life^5^^-^ 7. Despite the fact that a successful kidney transplant may reverse 2HPT in many cases, high levels of parathormone (PTH) may persist in some patients, a condition called persistent hyperparathyroidism after kidney transplant or tertiary hyperparathyroidism (3HPT). 3HPT injures the kidney graft and bone metabolism^8^.

In single parathyroid adenoma (1HPT), focal exploration directed by preoperative imaging and the intraoperative parathormone (ioPTH) monitoring is associated with high cure rates,^9^^,^ 10 as single gland disease is the main cause of hyperparathyroidism (HPT). By contrast with 1HPT, several parathyroid glands are affected in 2HPT and 3HPT. A significant reduction of glandular mass is necessary to avoid persistence.

The identification of four parathyroid glands is sufficient in the majority of patients with multiglandular disease. Nevertheless, in our experience, almost 12% of the cases had at least one supernumerary parathyroid gland^11^. These supernumerary glands are capable of maintaining high levels of PTH. The ioPTH sampling in 2HPT and 3HPT may help the surgeon avoid high PTH persistence by a supernumerary gland or a significantly hyperplastic missed ectopic parathyroid gland. Even in a bilateral exploration, ioPTH can suggest that additional search and resection are necessary.

In 2HPT and 3HPT, the reduction of 80% of ioPTH is a useful predictor of good outcome^12^. In addition to a higher cutoff, there is a standard recommendation to delay ioPTH sampling for at least 20 minutes^13^^,^ 14, due to renal patients' complexity. They have a multiglandular disease with asymmetric parathyroid proliferation. Besides that, PTH molecules and its fragments have altered reduction kinetics, resulting in a less predictable rate of ioPTH drop following the excision of hyperfunctioning tissue^15^^,^ 16.

The postponed sampling prolongs the surgical time, making ioPTH monitoring potentially not practicable. The present study had the main objective of verifying whether the ioPTH kinetics differs between patients with several renal functions. Additionally, it aimed to evaluate if delayed PTH sampling is necessary for dialysis and kidney transplant patients undergoing a PTx.

## Materials and methods

This was a retrospective study of ioPTH profile of patients undergoing PTx in a tertiary referral center (Clinics Hospital, University of Sao Paulo). The Institutional Review Board approved the study.

We reviewed the data of all patients with 2HPT on dialysis, and 3HPT and 1HPT undergoing surgical treatment from 2011 to 2015. Patients with less than 12 months of follow-up after surgery or who had surgical failure were excluded. 1HPT with double adenoma or multiglandular disease secondary to hyperplasia were also excluded.

The 2HPT group included some patients who were in a current randomized clinical trial developed at our institution that compared various PTx strategies (registered at Clinicaltrials.gov, NCT02464072). This primary study had three arms: subtotal PTx and two variants of total PTx with autograft. The operation type was assigned by randomization. Dialysis patients not participating in the study mentioned above underwent the treatment modality established in the institution: total PTx with autograft of 45 fragments of parathyroid tissue (TPTx-45). Patients with 3HPT underwent TPTx-45 or subtotal PTx according to surgeon discretion or based on prior discussion with the nephrology team. All patients with 1HPT underwent a focused-approach PTx based on preoperative localization imaging (ultrasonography and technetium^99mTc^ sestamibi scintigraphy).

We followed the patients according to a standardized surveillance schedule, which included laboratory tests at regular intervals (3, 6, and 12 months after surgery). Patients with 2HPT and 3HPT were classified into one of the possible therapeutic outcomes (success vs. failure) based on the occurrence of bone pain, and the upper limit of intact PTH, and calcium values recommended by the Kidney Disease: Improving Global Outcomes position statement^17^. For patients of the 3HPT group, PTH and serum calcium were in the normal range (15-65 pg/mL and 8.4-10.2 mg/dL, respectively). In the 2HPT group, PTH levels ranged from two to nine times the upper standard limit for the assay, and serum calcium was in the normal range. 1HPT classification was based on the PTH's values if they were below the upper limit of normality (65 pg/mL). Operation success was defined as reaching the therapeutic target in the first six months after surgery and maintaining it until the twelfth month. Surgical failure was not reaching the therapeutic target in the first six months after surgery.

At our institution, ioPTH samples are taken from the same internal jugular vein under direct view^18^. We used the following samples for the analysis: baseline ioPTH (sample taken at the beginning of the operation after access to the central neck compartment and prior to parathyroid exploration), a pre-excision sample (PE) taken before the excision of all identified parathyroid glands, ioPTH-10 min (10 minutes after excision of the parathyroid glands), and ioPTH-15 min (15 minutes after excision of the parathyroid glands).

Venous blood samples (3 to 5 mL) were collected in tubes without anticoagulants, immediately transported to the laboratory, and centrifuged at room temperature before analysis. The intact molecule ioPTH was analyzed by an electrochemiluminometric assay [CORELAB kits from ABBOT Laboratories (normal range 15 to 65 pg/mL)].

In our clinical practice, the results of ioPTH assays were used to confirm the adequate removal of the diseased parathyroid mass. A decrease ≥ 80% in the 10-minute sample compared to the highest baseline samples value (baseline or PE) was considered a surgical success predictor for patients in 2HPT and 3HPT groups, achieved in all operations (subtotal PTx or total PTx with autograft). For 1HPT, the desired target was a drop equal to or greater than 50% in the 10-minute sample. We performed the ioPTH-15 min to have a reserve sample in case of any laboratory problems or discrepant results with the first (ioPTH-10 min). Unsatisfactory level drop led to further dissection.

However, we used this research proposal to compare the decrease kinetic profile of patients with 1HPT, 3HPT, and 2HPT. The relative value or percent decrease was calculated considering the baseline sample as 100%. We compared both the ioPTH's absolute changes and percent decrease.

We reviewed the patient diagnosis, demographics, and pre- and postoperative laboratory values (intact PTH, total calcium, phosphorus, and creatinine). For 1HPT and 3HPT patients, creatinine clearance was estimated using the formula provided by the Chronic Kidney Disease Epidemiology Collaboration (available at https://sbn.org.br).

### Statistical analysis

Continuous variables were tested for normality with Kolmogorov-Smirnov test, and if they passed the test for normality, they were presented as mean ± standard deviation. Otherwise, nonparametric distributions were summarized as median and interquartile range (Q1-Q3). We present data as median and interquartile range in tables if the measure in one group was nonparametric. In variables with normal distributions, mean and median were very similar. For comparisons between 2 groups, we used Student t-test for parametric data and the Mann-Whitney test for nonparametric data. Accordingly, for inferential statistics, we considered the Kruskal-Wallis (with Dunn's Multiple Comparison Test) for nonparametric pairwise. Categorical variables are presented as count and frequency. The Chi-square and Fisher's exact test were employed to analyze these variables.

We used Spearman's correlation coefficient (r_s_) for non-parametric data.

Differences with a descriptive level (*P*) below 0.05 were considered significant.

## Results

Of the 256 PTx patients, sixteen were excluded. There were 57 cases with 1HPT, 162 with 2HPT, and 37 with 3HPT. Dialysis patients were significantly younger than 1HPT and 3HPT patients.

Demographic data and laboratory findings are shown in Table 1. Non-dialysis patients had similar preoperative PTH and total calcium serum values in 1HPT and 3HPT groups, regardless of HPT etiology, and whether there was a multiglandular (hyperplasia) or uniglandular (adenoma) lesion. Glomerular filtration rates ≥ 60 mL/min were found in 81.4% of the 1HPT group and in 48.4% of patients from the 3HPT group.

**Table 1:** Patient demographics and laboratory tests

	1HPT (n = 57)	2HPT (n = 162)	3HPT (n = 37)	p value
Preoperative laboratory tests				
Gender, F/M (%)	83.3/16.7	55.5/44.5	57.6/42.4	n/a
Intact parathormone (pg/mL), median (IQR)	161 (110-304)	1787 (1138-2187)	200 (126-405)	< 0.001*
				0.211^†^
				**< 0.001^‡^**
Total calcium (mg/dL), mean (SD)	10.9 (0.8)	9.6 (0.8)	10.9 (0.9)	< 0.001*
				0,936^†^
				**< 0.001^‡^**
Phosphorus, (mg/dL), mean (SD)	2.9 (0.9)	5.2 (1.5)	2.6 (1.2)	< 0.001*
				**0.011^†^**
				**< 0.001^‡^**
Creatinine, (mg/dL), median (IQR)	0.78 (0.64-0.96)	n/a	1.16 (1.02-1.70)	**< 0.001^†^**
Creatinine clearance, (mL/min/1.73 m^2^), mean (SD)	81 (28)	n/a	59 (25)	**< 0.001^†^**
Postoperative laboratory tests				
After a 12-month follow-up and successful outcome				
Intact parathormone (pg/mL), median (IQR)	47 (37-68)	78 (40-159)	62 (32-90)	0.002*
				0.395^†^
				0.05^‡^
Total calcium (mg/dL), mean (SD)	9.3 (0.5)	8.5 (1.0)	8.9 (1.0)	< 0.001*
				**0.046^†^**
				0.05^‡^
Phosphorus (mg/dL), mean (SD)	3.5 (0.6)	4.7 (1.5)	3.5 (0.9)	< 0.001*
				0.95^†^
				**< 0.001^‡^**
Creatinine (pg/mL), median (IQR)	0.8 (0.7-0.9)	n/a	1.3 (1.0-2.1)	**< 0.001^†^**
Creatinine clearance (mL/min/1.73 m^2^), mean (SD)	80 (27)	n/a	54 (29)	**< 0.001^†^**

F, female; M, male; IQR, interquartile range; SD, standard deviation; n/a, not applicable; Bold, p < 0.05.

* Comparing 1HPT and 2HPT groups.

† Comparing 1HPT and 3HPT groups.

‡ Comparing 2HPT and 3HPT groups.

Within the first year after PTx, some patients in the 1HPT and 3HPT groups had serum PTH levels above the upper limit of normality. These events occurred due to hungry bone syndrome, which reflects remineralization, or renal dysfunction (clearance of creatinine below 60 mL/min/1.73 m^2^).

**Figure 1:**
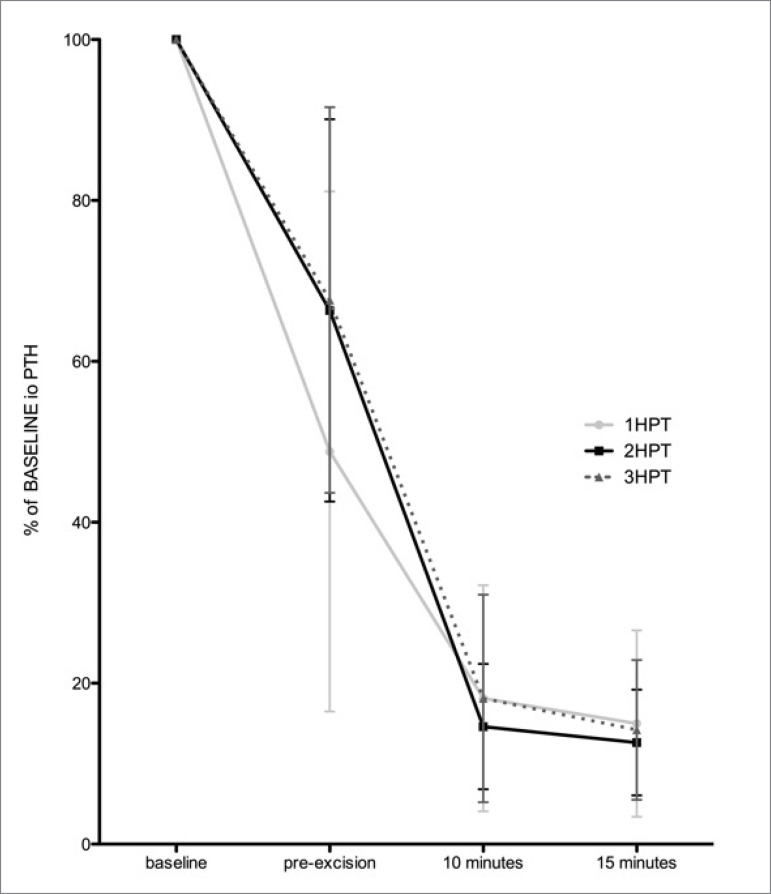
Mean and standard deviation of relative values of intraoperative parathormone (ioPTH) compared to baseline after successful parathyroidectomy in single parathyroid adenoma (1HPT), 2HPT (secondary hyperparathyroidism in dialytic patient), and 3HPT (persistent hyperparathyroidism after kidney transplant).

Absolute values of baseline ioPTH were significantly higher in 2HPT. Table 2 and Figure 1 present data from the three groups.

**Table 2:** Absolute and relative values of the intraoperative parathormone (median and interquartile range)

	1HPT (n=57)	2HPT (n=162)	3HPT (n=37)	p value
Age (years)	57 (53-68)	45 (34-54)	52 (44-59)	< 0.0001*^†^
Absolute baseline ioPTH (pg/mL)	218 (138-964)	1744 (1241-2738)	409 (298-660)	< 0.0001*^†^
Absolute PE ioPTH (pg/mL)	181 (100-380)	1371 (838-2054)	385 (230-878)	< 0.0001*^†^
Absolute ioPTH-10 min (pg/mL)	57 (38-94)	246 (170-344)	62 (40-102)	< 0.0001*^†^
Absolute ioPTH-15 min (pg/mL)	41 (29-68)	214 (156-292)	53 (32-80)	< 0.0001*^†^
Relative baseline ioPTH	100%	100%	100%	n/a
Relative ioPTH-10 min^a^ (%)	20 (10-31)	14 (11-19)	18 (10-21)	p = 0.143* ^†b^
Relative ioPTH-15 min^a^ (%)	16 (8-27)	12 (10 -16)	15 (8-18)	p = 0.07* ^†b^

n, (number of study patients); 1HPT, primary hyperparathyroidism; 2HPT, secondary hyperparathyroidism in dialytic patient; 3HPT, persistent hyperparathyroidism after kidney transplant; n/a, not applicable; Bold, p < 0.05.

* Comparing 2HPT and 1HPT groups.

† Comparing 2HPT and 3HPT groups.

a PTH compared to baseline.

b Not statistically significant.

In patients with successful PTx, the relative decreases in ioPTH were similar at 10 and 15 min after resection of hyperfunctioning parathyroid tissue, independent of the diagnosis. Beyond the tenth minute, the kinetics of ioPTH decline was similar between the groups.

We performed a correlation test to determine the relationship between creatinine clearance and percentual ioPTH decrease. A correlation between kidney function and relative decrease profile of ioPTH-10 min and ioPTH-15 min was not found (r_s_ = -0.028, *p* < 0.796 and r_s_ = -0.038, *p* < 0.729, respectively).

## Discussion

In the present study, the rate of ioPTH drop was not affected by kidney function from the tenth minute after parathyroid resection. Therefore, it is not necessary to delay sampling.

PTx remains a very important therapeutic tool in the management of all cases of HPT. Although new drugs added significant benefit to these patients,^19^ treatment refractoriness may occur when patients have severe morphological and functional changes in the parathyroid glands^20^. A successful PTx improves both the patient's quality of life and survival, while a persistent disease may affect all of these benefits. Remedial surgery entails increased risks and it can also fail. Therefore, intraoperative confirmation of the excised tissue by frozen section and ioPTH monitoring may represent useful tools in order to avoid residual hyperfunctioning tissue.

The monitoring of ioPTH is useful to suggest a supernumerary parathyroid gland. Eventually, in non-dialysis patients with a multiglandular disease (e.g., 3HPT or 1HPT due to multiple endocrine neoplasia syndromes) in which less than four parathyroid glands are found after cervical exploration, it may indicate satisfactory metabolic outcomes when associated with an ioPTH decrease into the established target. Nevertheless, the results of ioPTH monitoring are less straightforward than those of 1HPT^21^. The Miami criterion represents a reliable indicator to predict PTx success in 1HPT, as it indicates postoperative normocalcemia when the ioPTH-10 min value falls 50% or more, compared to the highest perioperatively sampled levels, after excision of the hyperfunctioning gland^22^^,^ 23. The same time-point reference of 10 min raises questions about the ioPTH measurement in the 2HPT and 3HPT.

In 2018, Egan et al.^24^ evaluated the influence of renal function deterioration on the kinetics of ioPTH decrease in a cohort of patients with 1HPT of uniglandular etiology and undergoing successful PTx. The authors reported significant differences only with severe renal function impairment, stage 4 and 5 of chronic kidney disease (CKD), primarily at 5 minutes after PTx, but that the kinetics started to equalize after 10 minutes, despite more slowly in renal patients.

Several studies about ioPTH monitoring in 2HPT initially proposed longer intervals between parathyroid resection and PTH sampling^12^^,^ 25 ^,^ 26. This was necessary due to metabolic specificities of these patients, who have a prolonged PTH half-life and accumulation of PTH fragments (7-84) that can cross-react in most laboratory tests (second-generation assay); these events can overestimate serum hormone levels.^27^

Matsuoka et al.^28^ reported in dialysis patients a 82.9% drop in ioPTH at 5 minutes, 90.7% at 10 minutes, 94.2% at 15 minutes, and 95.9% at 30 minutes, and they established predictive criteria of postoperative outcome, with high sensitivity and specificity at 10 minutes. The same opinion regarding definition of surgical success at 10 minutes is shared by Chou et al.^29^, after identifying a significant percentage decrease in ioPTH at 10 minutes (75.1% ± 6.2%). Their data suggest that it may not be necessary to wait long intervals for a reliable evaluation of ioPHP drop after parathyroid resection.

Unlike 1HPT, not all hormone levels return to normal during the 2HPT surgery, probably because of higher baseline PTH values in these patients^30^, resulting in a longer time for bloodstream elimination^29^. In our series, the dialysis group exhibited significantly higher serum levels of ioPTH in the different samples. In the intraoperative period, there was no drop to the standard reference values, even with surgical success-the patients in the transplanted group commonly normalized PTH levels within the tenth minute after PTx. The different metabolic conditions of the two situations justified this difference. Nevertheless, this did not interfere with the ioPTH's drop intensity after the 10 min reference point, since the relative decrease intensity had no statistical difference when comparing dialysis versus non-dialysis patients at 10 and 15 minutes after PTx.

In terms of metabolism, patients with 3HPT resemble those with 1HPT, presenting with hypercalcemia, reasonable renal function, and tendency to normalize serum PTH levels even intraoperatively. Nevertheless, from the structural point of view, 3HPT is a multiglandular disease (contrary to the typical disease that is uniglandular in the 1HPT), requiring different surgical approaches and probably interfering with the analysis of the ioPTH profile. This, together with the difficulty of assuming completely normal kidney function years after renal transplantation, leaves the transplant in an intermediate position with the dialytic rather than with the 1HPT^12^.

The ioPTH samples in the related CKD-HPT do not seem to make the surgery more time-saving, even with the early ioPTH-10 min pattern, and seem not cost-effective. However, the relatively low cost of ioPTH monitoring and the association between persistent HPT and higher cardiovascular events, for which hospital admissions are very costly to the health system, make the ioPTH a justifiable cost-benefit tool to avoid HPT persistence.

The present study has an important limitation. Due to the aim and design, it was not possible to evaluate the real benefits of ioPTH monitoring in 2HPT and 3HPT. Future studies using a homogeneous series are necessary to verify whether the ioPTH-10 min monitoring helps predict surgical outcome in patients with related CKD-HPT and evaluate the impact of decision-making during surgery. Additionally, it is essential to find a pattern that differentiates failure from surgical success, and test the accuracy of the method.

In conclusion, the ioPTH sampling routine should not be changed in 2HPT and 3HPT when compared to that of 1HPT, as the ioPTH decrease rate drops significantly at 10 minutes. Delayed sampling appears to be unnecessary in dialysis and kidney transplant patients undergoing a PTx, irrespective of the cut-off value adopted.
